# Health Seeking Behaviour among Individuals with Presumptive Tuberculosis in Zambia

**DOI:** 10.1371/journal.pone.0163975

**Published:** 2016-10-06

**Authors:** Pascalina Chanda-Kapata, Nathan Kapata, Felix Masiye, Mwendaweli Maboshe, Eveline Klinkenberg, Frank Cobelens, Martin P. Grobusch

**Affiliations:** 1 Department of Disease Surveillance, Control and Research, Ministry of Health, Lusaka, Zambia; 2 Center of Tropical Medicine and Travel Medicine, Department of Infectious Diseases, Academic Medical Centre, University of Amsterdam, Amsterdam, Netherlands; 3 National TB/Leprosy Control Program, Department of Disease Surveillance, Control and Research, Ministry of Health, Lusaka, Zambia; 4 Department of Economics, University of Zambia, Lusaka, Zambia; 5 World Health Organization Country Office, Lusaka, Zambia; 6 Epidemiology, KNCV Tuberculosis Foundation, The Hague, Netherlands; 7 Department of Global Health and Development, Academic Medical Centre, Amsterdam, Netherlands; University of Cape Town Lung Institute, SOUTH AFRICA

## Abstract

**Background:**

Tuberculosis (TB) prevalence surveys offer a unique opportunity to study health seeking behaviour at the population level because they identify individuals with symptoms that should ideally prompt a health consultation.

**Objective:**

To assess the health-seeking behaviour among individuals who were presumptive TB cases in a national population based TB prevalence survey.

**Methods:**

A cross sectional survey was conducted between 2013 and 2014 among 66 survey clusters in Zambia. Clusters were census supervisory areas (CSAs). Participants (presumptive TB cases) were individuals aged 15 years and above; having either cough, fever or chest pain for 2 weeks or more; and/or having an abnormal or inconclusive chest x-ray image. All survey participants were interviewed about symptoms and had a chest X-ray taken. An in-depth interview was conducted to collect information on health seeking behaviour and previous TB treatment.

**Results:**

Of the 6,708 participants, the majority reported at least a history of chest pain (3,426; 51.1%) followed by cough (2,405; 35.9%), and fever (1,030; 15.4%) for two weeks or more. Only 34.9% (2,340) had sought care for their symptoms, mainly (92%) at government health facilities. Of those who sought care, 13.9% (326) and 12.1% (283) had chest x-ray and sputum examinations, respectively. Those ever treated for TB were 9.6% (644); while 1.7% (114) was currently on treatment. The average time (in weeks) from onset of symptoms to first care-seeking was 3 for the presumptive TB cases. Males, urban dwellers and individuals in the highest wealth quintile were less likely to seek care for their symptoms. The likelihood of having ever been treated for TB was highest among males, urban dwellers; respondents aged 35–64 years, individuals in the highest wealth quintile, or HIV positive.

**Conclusion:**

Some presumptive TB patients delay care-seeking for their symptoms. The health system misses opportunities to diagnose TB among those who seek care. Improving health-seeking behaviour among males, urban dwellers and those with a higher social economic status; and addressing health care lapses in TB case detection is required if TB is to be effectively controlled in Zambia.

## Introduction

Zambia records approximately 43,000 tuberculosis (TB) cases annually [1], with an estimated prevalence of 455/100,000 [2] for all forms of TB in the country. The target of any TB control program is to detect and treat incident cases early. This is because timely diagnosis and treatment of TB has individual and community benefits in curbing personal ill health and transmission of the disease [3]. This is, however, difficult to achieve because patients usually present late to the health facility and when they do, health care workers may fail to diagnose TB among symptomatic individuals [4]. The reasons for delays in health-seeking for TB related symptoms are due to both patient and provider factors; such as fear of social isolation, economic constraints, distance to the health facility, inadequate staff, attitudes and poor quality of health services [3, 5]. Stigma towards TB patients may be another cause for health seeking delays, as it has been demonstrated in studies conducted among TB patients in Africa and Asia [6,7,8]. The health-seeking behaviour of TB patients has been demonstrated to be affected by the patient’s socioeconomic circumstances in studies conducted at diagnostics centres or clinics in various countries [9–15]. Delays from the health care provider side in diagnosing and starting treatment have also been documented in Asia and Africa [10,16].

Health-seeking behaviour of presumptive TB patients in Zambia has been studied only at health facility level [12, 17]. To our knowledge, this is the first population-based study to assess health-seeking behaviour of presumptive TB cases and previously treated TB patients in Zambia. TB prevalence surveys offer a unique opportunity to study health seeking behaviour at the population level because they identify individuals with symptoms that should ideally prompt a health consultation, and should trigger health services to exclude TB–irrespective of whether they have TB or not. Additionally, they allow studying health-seeking behaviour for TB symptoms among patients previously treated for TB, who may be more inclined to seek TB care. Therefore, we assessed health-seeking behaviour of presumptive TB cases for TB symptoms as part of a nationwide TB prevalence survey conducted in Zambia. Specifically, we sought to identify proportions of participants who sought care; previous TB treatment history and their provider choice. Areas for potential programme improvement are discussed.

## Materials and Methods

### Study design

This was a cross-sectional study of health-seeking behaviour of individuals with signs and symptoms suggestive of TB, and of their provider choice. The study population was drawn from the national TB prevalence survey, a nationwide survey of 66 clusters, sampled proportionally to size, and representing both rural and urban Zambia. The primary sampling unit (cluster) was a census supervisory area (CSA).

### Study population

All survey participants (aged 15 years and older who were residents of the survey clusters) in the national TB prevalence survey were interviewed about symptoms and had a chest X-ray taken.

### Data collection

Individuals who were TB survey participants underwent symptom screening for cough or chest pain or fever lasting 2 weeks or more. Thereafter, chest x- ray screening for abnormalities consistent with TB was performed. Those who were found to have one or more of the symptoms and or any abnormal/inconclusive chest x-ray were eligible for sputum submission for TB diagnosis and included in the present analysis as ‘presumptive TB cases’. The presumptive TB cases underwent a detailed in-depth interview using a standardized structured questionnaire on symptoms, time of disease onset, health-seeking behavior, treatment history and health care access issues. In addition, all survey participants were offered HIV antibody testing.

### Data analysis

Data was analysed using STATA version 12. Frequencies with their 95% confidence intervals were estimated for the outcomes of interest. Wealth quintiles were generated by principal components analysis using a standard list of household assets and access to basic amenities. Both univariate and multivariate logistic regression analysis was performed in order to determine the relationship between outcome variables and participant characteristics. Kruskal-Wallis equality-of-populations rank test was performed for non-parametric outcomes (time from onset of symptoms to first care-seeking).

### Ethics statement

The study protocol was cleared by the University of Zambia’s Biomedical Research Ethics Committee (UNZABREC), approval number 020-08-12. Authorisation to conduct the survey was sought in line with the existing national policies and guidelines at national, provincial and district levels. Written informed consent was obtained from all individuals who agreed to participate in the survey. For minors, both the assent of the minor and the consent of the next of kin or caretakers or guardian were obtained in writing for each participant aged 15–17 years. The Institutional Review Board (IRB) approved this consent procedure. All the consent or assent forms were recorded on standard forms which were developed for the study and these were filed in lockable cabinets at the end of each cluster operation.

## Results

**Fig 1:**
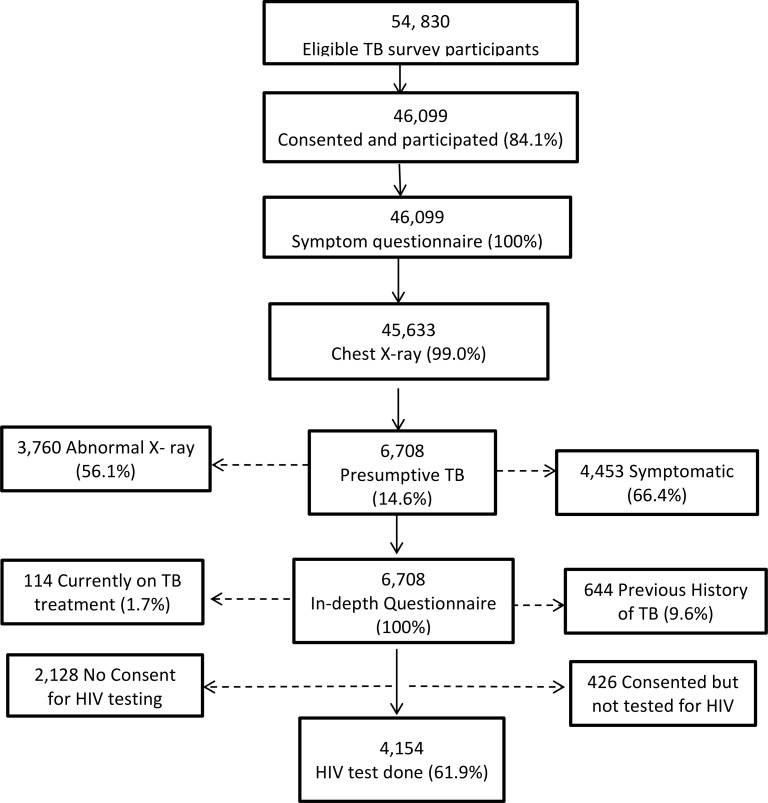
Participant flow chart.

There were 54,830 participants who were eligible to participate in the TB survey and 46,099 (84.1%) gave consent and turned up for investigations. All 46,099 (100%) underwent symptom screening to identify history of chest pain, cough or fever. Subsequently, 45,633/46,099 (99.0%) underwent field level chest x-ray screening. Based on symptom and or chest x-ray screening, 6,708 (14.6%) participants were classified as presumptive TB cases and included in the present analysis. Fig 1 outlines the flow of participants.

The general description of the background characteristics of the presumptive TB cases are presented in Table 1. The majority were from a rural setting (67%) and had some primary education (52%); 23% were aged more than 65 years; 22% were from the lowest wealth quintile and the HIV positivity among those tested was 6.2%.

**Table 1:** Presumptive TB symptoms, health seeking and treatment history.

Variable	Presumptive TB cases N (%)	Chest pain > = 2 weeks N (%)	Cough> = 2 weeks N (%)	Fever> = 2 weeks N (%)	Sought care for symptoms N(%)	Ever treated for TB N (%)
**Total**	6708	3426 (51.07)	2405 (35.9)	1030 (15.4)	2340 (66.4)	644 (9.6)
**Age group**						
15–24	716 (10.7)	386 (11.3)	333(13.8)	147 (14.3)	192 (8.2)	26 (4.0)
25–34	1,051 (15.7)	570 (16.6)	403 (16.8)	169 (16.4)	383 (16.4)	87 (13.5)
35–44	1,249 (18.6)	685 (20.0)	434 (18.1)	194 (18.8)	410 (17.5)	158 (24.5)
45–54	1,100 (16.4)	563 (16.4)	363 (15.1)	161 (15.6)	407 (17.4)	142 (22.1)
55–64	1,019 (15.2)	502 (14.7)	327 (13.6)	116 (11.3)	370 (15.8)	120 (18.6)
65+	1,573 (23.4)	720 (21.0)	545 (22.7)	243 (23.6)	578 (24.7)	111 (17.2)
**Sex**						
Male	3363 (50.1)	1542 (45.0)	1251(52.0)	516 (50.1)	1055 (45.1)	378 (58.7)
Female	3345 (49.9)	1884 (55.0)	1154(48.0)	514 (49.9)	1285 (54.9)	266 (41.3)
**Setting**						
Rural	4,471 (66.7)	2641(77.1)	1741(72.4)	756 (73.4)	1740 (74.4)	327(50.8)
Urban	2,237 (33.3)	785 (22.9)	664 (27.6)	274 (26.6)	600 (25.6)	317 (49.2)
**Wealth Quintile(N = 5,930)**						
Lowest	1,323 (22.3)	772 (29.0)	540 (25.7)	254(27.5)	512 (24.8)	94 (15.8)
Second Lowest	1,119 (18.9)	687 (25.8)	433 (20.6)	202(21.9)	445 (21.6)	71 (11.9)
Middle	1,258 (21.2)	666 (25.0)	450 (21.4)	210 (22.7)	516 (25.0)	128 (21.4)
Fourth	1,179 (19.9)	514 (19.3)	383(18.2)	147 (15.9)	339 (16.4)	145 (24.3)
Highest	1,051 (17.7)	351 (13.2)	297 (14.1)	111 (12.0)	252 (12.2)	159 (26.6)
**Education Level**						
None	1,159 (17.3)	643 (18.8)	410 (17.0)	173 (16.8)	481 (20.6)	81 (12.6)
Primary	3,463 (51.6)	1873 (54.7)	1287 (53.5)	591(57.4)	1229 (52.5)	275 (42.7)
Secondary	1,903 (28.4)	842 (24.6)	669 (27.8)	251(24.4)	582(24.9)	262(40.7)
Tertiary	182 (2.7)	67(2.0)	39 (1.6)	15(14.6)	47 (2.0)	26 (4.0)
Unknown	1 (0.0)	1(0.0)	0 (0.0)	0 (0.0)	1 (0.0)	0 (0.0)

Of the 6,708 presumptive TB cases 4,453 (66.4%) reported, one or more of the three screening symptoms while 3,760 (56.1%) had abnormal or inconclusive X-ray; 2,948 (43.9%) were detected by symptoms alone; 2,255 (33.6%) by abnormal or inconclusive chest x-ray; and 1,505 (22.4%) by both symptoms and chest x-ray. At the time of the survey, 114 (1.7%) of the presumptive cases were on TB treatment. A total of 644 presumptive cases (9.6%) reported a previous history of TB treatment (Table 1), while 636 (9.5%) indicated that a member of their household had been treated for TB prior to the survey.

Of the 2,340 (34.9%) presumptive TB individuals who sought care, the majority (81.8%) had chosen a government facility for their first visit (Table 2). The males were less likely than females to use a government health facility (RR = 0.43; p = 0.004); those aged 25 years and above were more likely to use a government health facility than those aged 15–24 years (RR = 1.52; p = 0.000); the urban residents were less likely than their rural counter parts to uses a government facility (RR = 0.64; p = 0.000); individuals from the highest wealth quintiles were less likely to choose a government facility for their first visit than those from the lowest wealth quintile (RR 0.49; p = 0.000) and the educated were less likely than the uneducated to use a government health facility (p<0.05). Almost 326 (13.9%) had a chest X-ray performed and 283 (12.1%) reported having had a sputum smear examination.

**Table 2:** Seeking care, smear and chest x-ray examination by provider type.

First provider type	Sought care for symptoms N (%)	Sputum smear done N (%)	Chest x-ray taken N (%)
**Government/community clinic**	1915 (81.8)	219 (77.4)	232 (71.2)
**Private Clinic/hospital**	85 (3.6)	7 (2.5)	12 (3.7)
**Government Provincial/district hospital**	228 (9.7)	40 (14.1)	62 (19.0)
**Pharmacy**	24 (1.0)	5 (1.8)	4 (1.2)
**Private doctor**	3 (0.1)	0	0
**Traditional healer**	1 (0.0)	n/a	n/a
**Faith based organization**	55 (2.4)	7 (2.5)	10 (3.1)
**Other**	29 (1.2)	5 (1.8)	6 (1.8)
**Total**	**2340**	**283**	**326**

The average time (in weeks) from onset of symptoms to first care-seeking was 3; with majority (32%) of the cases seeking care 2–3 weeks from onset of symptoms (p = 0.0001). The distribution of those who sought care by time from onset of symptoms to seeking care is shown in Table 3.

**Table 3:** Time from onset of symptoms to first seeking care.

Time from onset of symptoms to first seeking care (weeks)	Frequency (%)	Binomial Exact 95% CI
**0–1**	640 (27.4)	25.6–29.2
**2–3**	753 (32.2)	30.3–34.1
**4–5**	394 (16.8)	15.3–18.4
**6+**	553 (23.6)	21.9–25.4
**Total**	2340 (100)	

Table 4 shows the odds of seeking care by background characteristics of the presumptive TB cases. The older age groups were 1.6 times more likely to seek care than the younger. The males and urban dwellers were less likely to seek care than their female and rural counterparts, (OR = 0.8 and 0.6 respectively). Presumptive TB cases in two highest wealth fourth and fifth wealth quintiles were less likely to seek care for their TB symptoms than those from the lowest wealth quintiles (OR = 0.7 and 0.5, respectively). The educated were less likely to seek care than their uneducated counterparts.

**Table 4:** Univariate and multivariate association of having sought care for tuberculosis symptoms with age, sex, setting, wealth, education level and HIV infections status.

Variable	Sought care N (%)	Univariate logistic regression		Multivariate logistic regression	
		Odds ratio (95% CI)	p-values	Odds ratio (95%CI)	p-values
**Age group**	** **	** **	** **	** **	** **
15–24	192 (8.2)	Ref			
25–34	383 (16.4)	1.62(1.32–1.98)	0	1.61 (1.31–1.99)	0
35–44	410 (17.5)	1.39(1.14–1.69)	0.001	1.37 (1.11–1.68)	0.003
45–54	407 (17.4)	1.70 (1.39–2.08)	0	1.67 (1.36–2.06)	0
55–64	370 (15.8)	1.76 (1.43–2.15)	0	1.66 (1.35–2.05)	0
65+	578 (24.7)	1.67(1.38–2.01)	0	1.64(1.35–2.00)	0
**Sex**	** **	** **	** **	** **	** **
Female	1,285 (54.9)	Ref			
Male	1,055 (45.1)	0.81 (0.74–0 .90)	0	0.75 (0.68–0.83)	0
**Setting**					
Rural	1,740 (74.4)	Ref			
Urban	600 (25.6)	0.68 (0.62–0 .76)	0	0.60 (0.53–0.67)	0
**Wealth quintile**					
Lowest	512 (24.8)	Ref			
Second Lowest	445 (21.6)	1.03 (0.88–1.21)	0.69	1.04 (0.88–1.23)	0.625
Middle	516 (25.0)	1.15 (0.98–1.34)	0.087	1.12 (0.96–1.32)	0.15
Fourth	339 (16.4)	0.78 (0.66–0.91)	0.002	0.67 (0.57–0.80)	0
Highest	252 (12.2)	0.67 (0.56–0.79)	0	0.53 (0.44–0.64)	0
**Education**					
None	481 (20.6)	Ref			
Primary	1,229 (52.5)	0.79 (0.69–0 .91)	0.001	0.76 (0.68–0.89)	0
Secondary	-24.9	0.72 (0.62–0.84)	0	0.64 (0.55–0.75)	0
Tertiary	47 (2.0)	0.76 (0.55–1.05)	0.093	0.55 (0.39–0.80)	0.001
Unknown	1 (0.0)	empty			
**HIV Status**					
Negative	1,325 (56.6)	Ref			
Positive	161 (6.9)	1.20 (0.97–1.47)	0.086	1.18 (0.95–1.46)	0.127
Indeterminate	7 (0.3)	0.77 (0.31–1.91)	0.568	0.73 (0.28–1.90)	0.517
No consent	743 (31.8)	1.11 (0.99–1.23)	0.071	1.02 (0.91–1.14)	0.721

A history of TB treatments was 3–4 times more likely among presumptive TB cases aged 35–64 years than among those aged 15–24 years (Table 5). However, the odds were about twice for those aged 25–34; or 65 years and above respectively, than those aged 15–24 years. The odds of prior TB treatment was 1.5 times higher among male than among female presumptive TB cases; and 2 times higher in the urban than in the rural population. Individuals in the higher wealth quintile and those well-educated were more likely to have been treated for TB than those in the lowest quintile and with no formal education, respectively. The likelihood of having a history of TB treatment was 3 times higher among the HIV-positive than the HIV-negative individuals.

**Table 5:** Univariate and multivariate association of a history of previous TB treatment with age, sex, setting, wealth, education level and HIV infections status.

Variable	Ever treated for TB N (%)	Univariate logistic regression		Multivariate logistic regression	
		Odds ratio (95% CI)	p-values	Odds ratio (95% CI)	p-values
**Age group**	** **	** **	** **		
15–24	26 (4.0)	Ref			
25–34	87 (13.5)	2.30 (1.49–3.56)	0	2.40 (1.53–3.75)	0
35–44	158 (24.5)	3.61 (2.39–5.45)	0	3.84 (2.51–5.88)	0
45–54	142 (22.1)	3.73 (2.46–5.66)	0	3.93 (2.56–6.05)	0
55–64	120 (18.6)	3.37 (2.21–5.15)	0	3.54 (2.29–5.48)	0
65+	111 (17.2)	1.94 (1.27–2.96)	0.002	2.01 (1.30–3.12)	0.002
**Sex**					
Female	266 (41.3)	Ref			
Male	378 (58.7)	1.39 (1.18–1.64)	0	1.46 (1.24–1.72)	0
**Setting**					
Rural	327(50.8)	Ref			
Urban	317 (49.2)	2.05 (1.74–2.41)	0	2.09 (1.76–2.46)	0
**Wealth quintile**					
Lowest	94 (15.8)	Ref			
Second Lowest	71 (11.9)	0.87(0.64–1.19)	0.375	0.88 (0.64–1.22)	0.449
Middle	128 (21.4)	1.38(1.05–1.82)	0.02	1.47 (1.12–1.95)	0.006
Fourth	145 (24.3)	1.71(1.31–2.23)	0	1.82 (1.39–2.40)	0
Highest	159 (26.6)	2.17(1.67–2.83)	0	2.32 (1.77–3.04)	0
**Education**					
None	81 (12.6)	Ref			
Primary	275 (42.7)	1.14(0.88–1.46)	0.316	1.15 (0.89–1.48)	0.294
Secondary	262(40.7)	2.04(1.58–2.63)	0	2.12 (1.63–2.75)	0
Tertiary	26 (4.0)	2.11(1.32–3.37)	0.002	2.21 (1.38–3.55)	0
Unknown	-	empty			
**HIV Status**					
Negative	266 (41.3)	Ref			
Positive	68 (10.6)	2.52 (1.90–3.36)	0	2.56 (1.92–3.42)	0
Indeterminate	19(3.0)	2.10 (0.61–7.16)	0.238	2.16(0.62–7.38)	0.219
No consent	291(45.2)	2.05 (1.72–2.44)	0	2.06 (1.73–2.46)	0

## Discussion

This is the first nationally representative population-based survey to document health-seeking behaviour among presumptive and confirmed TB cases in Zambia.

In this study, only 35% of the presumptive TB cases reported to have sought care for their symptoms. Of those who had sought care, the majority had chosen a public health facility. Additionally, less than a quarter of those who sought care had sputum or chest x-ray examinations. The average time from onset of symptoms to first time seeking care was found to be around three weeks for the majority of presumptive TB cases. Males, younger age groups, urban dwellers and those from higher wealth quintiles were less likely to seek care for their TB symptoms. The poor and less educated were less likely to have had prior TB treatment. However, the HIV positive were 3 times more likely to have had prior TB treatment.

Our findings suggest that among Zambian presumptive TB cases, some delays in health care-seeking do occur. However, even for patients who do seek care, the opportunity to make a TB diagnosis at the health facility can be missed. The initial health provider choice was found to be appropriate in the majority (>90%) of patients who sought care since a medical facility (health centre) was a source of care. Unfortunately, the overall effectiveness of the appropriate provider choice is negated by the fact that this did not always translate into a TB diagnosis being made. This highlights the need to intensify the behaviour change communication for both patients and health care providers. In a qualitative study at an urban clinic in Zambia, TB-related stigma was perceived to be high, with negative consequences disproportionately higher among women than men [18]. Stigma towards TB has been widely reported elsewhere [6,7,8] and is known to affect the patient decision to seek care. Therefore, effective strategies of addressing TB stigma will be require to be tested so as to address patient delays in seeking care.

It was observed that the delay in seeking care was about three weeks, irrespective of the nature of symptoms. In this period, the individuals can transmit the infection to other community members. It is therefore important to put strategies in place to reduce patient delay so that transmission in the community can be reduced, and those with the disease can be put on treatment [19].

Previous TB treatment history was found to be more likely in the older, especially those aged 35–64 years. Additionally, those belonging to the highest wealth quintile and being well-educated were more likely to have been treated for TB, compared to those in the lowest quintile and with no education, respectively. It is possible that access to TB diagnosis and treatment may be better in these sub-groups. Additionally, since HIV prevalence is higher among these groups and HIV positivity was associated with a higher likelihood of a history of TB treatment, it is possible that HIV care may have provided an opportunity for TB detection and treatment. Nonetheless, the causes of these disparities need to be further investigated and addressed appropriately because it has been pointed out that health seeking consists of the interplay of both supply and demand side factors [20]. Therefore, strategies such as TB/HIV integration targeting the marginalised and improving health worker capacity to make a TB diagnosis may offer a solution to effectively detect and treat incident TB cases in Zambia.

The occurrence of chest pain was higher than cough and fever in both the presumptive and confirmed TB groups. However, the majority of those with cough more than 2 weeks were found to be TB patients. Therefore, a cough of two weeks or more may generally be more specific at predicting TB, as has been stated in other studies [21, 22]. Nonetheless, efforts to rule out TB should be made among individuals presenting with chest pain. This can be an important entry point for collaborating with cardiac related programs as chest pains may be more associated with cardiac problems [23].

The public sector still has a larger role to play in TB diagnosis in Zambia, as the majority of the participants who sought care chose a government-owned facility. However, a number of people sought care through the private and mission facilities as well. Thus, public-private partnerships are an important avenue to explore in order to create more options for access to TB care services, according to the patient situation and preferences, and more so in the urban areas where private providers are growing in number [24].

The patient delay found in this study is shorter than what was found in rural hospitals in Nigeria [25], rural South Africa [4] and a referral hospital in Kenya [15], but more than what was found in an urban clinic in Zambia [12]. However, the patient delay was similar to what was found in Vietnam [13]. It must be noted here that the time from onset of symptoms to first seeking care may have been affected by recall bias. Still, the Zambia National Tuberculosis Program (NTP) does not specifically define the patient delay threshold. There is need for the NTP to clearly define the threshold for delays and disseminate such information to both the patients and health care providers.

## Conclusion

There are supply and demand side factors affecting health care-seeking for TB in Zambia. Some presumptive patients delay seeking care for their symptoms. However, patients with TB symptoms are missed even when they seek care, and the majority seeks care through public health facilities. Targeted strategies to improve health seeking among males, urban dwellers and those with a higher social economic status are required. Future TB control strategies should address these patient delays and health care lapses if TB is to be effectively controlled in Zambia.

Information on the reasons for individuals not seeking care and the source of medication for those currently on treatment was not collected. Future surveys should endeavour to collect this information which has potential to highlight areas of program improvement.
